# Post-liver transplantation bacterial infection: current status, prevention and treatment

**DOI:** 10.3389/fcimb.2025.1698937

**Published:** 2025-11-25

**Authors:** Jingjie Guo, Ying Wang, Jingmiao Ma, Yiran Xu, Baojie Shi, Wenbin An, Jie Wang, Hao Li

**Affiliations:** 1 Organ Transplantation Clinical Medical Center of Xiamen University, Department of General Surgery, Xiang’an Hospital of Xiamen University, School of Medicine, Xiamen University, Xiamen, China; 2 Organ Transplantation Institute of Xiamen University, Xiamen Human Organ Transplantation Quality Control Center, Xiamen Key Laboratory of Regeneration Medicine, Xiamen, China; 3 Fujian Provincial Key Laboratory of Organ and Tissue Regeneration, School of Medicine, Xiamen University, Xiamen, China

**Keywords:** liver transplantation, bacterial infection, bacterial infections, post-liver transplantation, treatment

## Abstract

Liver transplantation (LT) is one of the most effective treatments for end-stage liver disease, as evidenced by a 1-year survival rate of approximately 90% and a 5-year survival rate exceeding 70%. Bacterial infections not only are major complications affecting the quality of life and graft function of LT patients but also constitute the primary causes of morbidity and mortality in this population. Additionally, the rejection response following LT increases the need for postoperative immunosuppressive therapy, and because of the complexity of the immune response in both donors and recipients, LT recipients are more susceptible to bacterial infections than other postoperative patients are. Reports indicate that gram-negative bacteria (such as *Enterobacter*, *Klebsiella*, and *Pseudomonas*) and gram-positive bacteria (such as *Staphylococcus* and *Enterococcus*) are common pathogens causing infections after LT. In particular, LT patients are prone to infection with multidrug-resistant (MDR) bacteria, which further complicates infection management. New detection technologies (such as digital droplet PCR, high-resolution melting, surface-enhanced Raman spectroscopy, and cell-mediated immunity) are highly sensitive in the early identification of drug-resistant bacteria and assessment of graft damage. Combining perioperative antibiotic and nonantibiotic therapy can help prevent infections and improve patient prognosis. Currently, effective precautionary warning systems are still lacking internationally, and issues such as dysbiosis caused by broad-spectrum antibiotics and overreliance on traditional methods for infection diagnosis and treatment need to be urgently addressed. This article reviews the relevant literature on the epidemiology and causes of post-LT bacterial infections and new diagnostic and treatment methods to provide a reference for the clinical prediction and prevention of such infections.

## Introduction

Bacterial infection after liver transplantation (LT) is a significant factor leading to patient mortality, particularly in the early postoperative period (Lacaille, 2012; Vilarinho and Lifton, 2012; Pedersen and Seetharam, 2014; Weiss and Thabut, 2019). The primary pathogens involved in post-LT infections include gram-negative bacteria such as *Pseudomonas aeruginosa* and *Escherichia coli*, as well as gram-positive bacteria such as *Staphylococcus aureus* and *Enterococcus* spp (del Pozo, 2008; Martin-Gandul et al., 2015; Hand and Patel, 2016; Idossa and Simonetto, 2017). In recent years, the misuse of antibiotics has led to the emergence of multidrug-resistant (MDR) organisms, which are closely associated with increased mortality rates in patients with post-LT infections (Kusne et al., 1988; Patel and Paya, 1997; Singh et al., 2004; Reid et al., 2009; van Delden, 2014; Righi, 2018). The unique characteristics of LT patients, including preoperative comorbidities, the complexity of the surgical procedure, postoperative immunosuppressive therapy, and frequent hospitalizations, increase the likelihood of bacterial infections (Bennett et al., 2015). The aim of this review is to explore the current status of research on the primary sites, risk factors, detection methods, infection mechanism and prevention and treatment strategies for bacterial infections after LT.

## Current status of bacterial infections

### Pathogen profile

Research indicates that 40% to 89% of LT patients experience varying degrees of infection within the first year after LT (Rayes et al., 2005; van Delden, 2014; Martin-Gandul et al., 2015; Diaz et al., 2024; Zuccaro et al., 2024). Among these, bacterial infections are the most common, accounting for 30% to 60% of all cases (Fishman, 2007; Gelb and Feng, 2009; Kawecki et al., 2009; Baganate et al., 2018; Righi, 2018; Ferrarese et al., 2024; Wu et al., 2024). During the first month after LT, most bacterial infections are related to prolonged surgical duration, massive blood loss during surgery, and the induction of immunosuppression (IS) at high doses (Shen et al., 2007; Shinde and Kapoor, 2024). From the second to the sixth months after LT, bacterial infections are frequently multifactorial in that they include immunosuppressive therapy; persistent infections from the perioperative period, such as *Clostridium difficile* colitis; residual pneumonia; technical issues (e.g., anastomotic leaks, empyema, cholangitis, and infected hematoma) (Fishman and Rubin, 1998; Fishman, 2007; Fishman, 2017); the cumulative effect of preoperative colonization by opportunistic pathogens; and maintenance IS with periodic modification on the basis of graft function. Bacterial infections occurring more than six months post-transplantation are associated with environmental exposure, late biliary complications, graft function, reactivation of hepatitis viruses, maintenance of low-key IS levels, and community exposure (Cervera et al., 2011).

The pathogens present after LT vary across different geographic regions. In North America (Safdar et al., 2004; Hellinger et al., 2009; Reid et al., 2009; Lee et al., 2011b; Shoji et al., 2015; Viehman et al., 2016; Anesi et al., 2018; Rolak et al., 2024), *Enterococcus* spp. are the most frequently isolated pathogens (31–42%), followed by *E. coli* (15–24%). In Europe (Moreno et al., 2007; Garcia Prado et al., 2008; Bert et al., 2010; Karvellas et al., 2011; Beranger et al., 2020; Hrenczuk et al., 2020; Rasmussen et al., 2021; Kremer et al., 2022; Neofytos et al., 2023; Martin-Mateos et al., 2024), gram-positive bacteria, such as *Enterococcus* spp., *S. aureus*, and methicillin-resistant coagulase-negative *staphylococci* (MRCNS), are present in the early period after transplantation, whereas gram-negative bacteria, such as *Enterobacter* spp., become predominant later.

In South America (Freire et al., 2013; Santoro-Lopes and de Gouvea, 2014; Oliveira et al., 2019; Freire et al., 2021; Lemos et al., 2024), *Klebsiella pneumoniae* is the most common pathogen that causes surgical site infections (SSIs), which may be related to inappropriate postoperative antibiotic treatment and insufficient nosocomial infection prevention measures.

In contrast, Asian (Avkan-Oguz et al., 2015; Aktas et al., 2019; Pouladfar et al., 2019; Jafarpour et al., 2020; Selimoglu et al., 2020) LT patients exhibit a higher rate of gram-negative bacterial infections caused by *Acinetobacter* spp. and *Klebsiella* spp. and a higher rate of multidrug resistance. In China and South Korea (Shi et al., 2009; Kim et al., 2022; Zhao et al., 2022; Liu et al., 2023; Tu et al., 2023; Wu et al., 2023; Choi et al., 2024; Sun et al., 2024), *Enterococcus faecium* is the dominant gram-positive bacterium, and *K. pneumoniae* (17/90, 18.9%) is the most common gram-negative bacterium detected in LT patients. In Japan (Iida et al., 2010), the most common pathogen is *S. aureus*, “methicillin-resistant S. aureus” (MRSA), followed by *Klebsiella* spp. and MRCNS. In the Philippines (Abad et al., 2017), *Enterococcus* spp. are the pathogens most frequently identified. Among all the detected bacteria, *Staphylococcus* spp. (34.3%) and methicillin-resistant MRCNS (43.2%) are the dominant species and multidrug-resistant organisms, respectively.

In Oceania (Tun et al., 2024), the most common microorganisms isolated from SSIs are MRCNS, followed by *Enterococcus* spp. and *Corynebacterium*.

**Figure 1:**
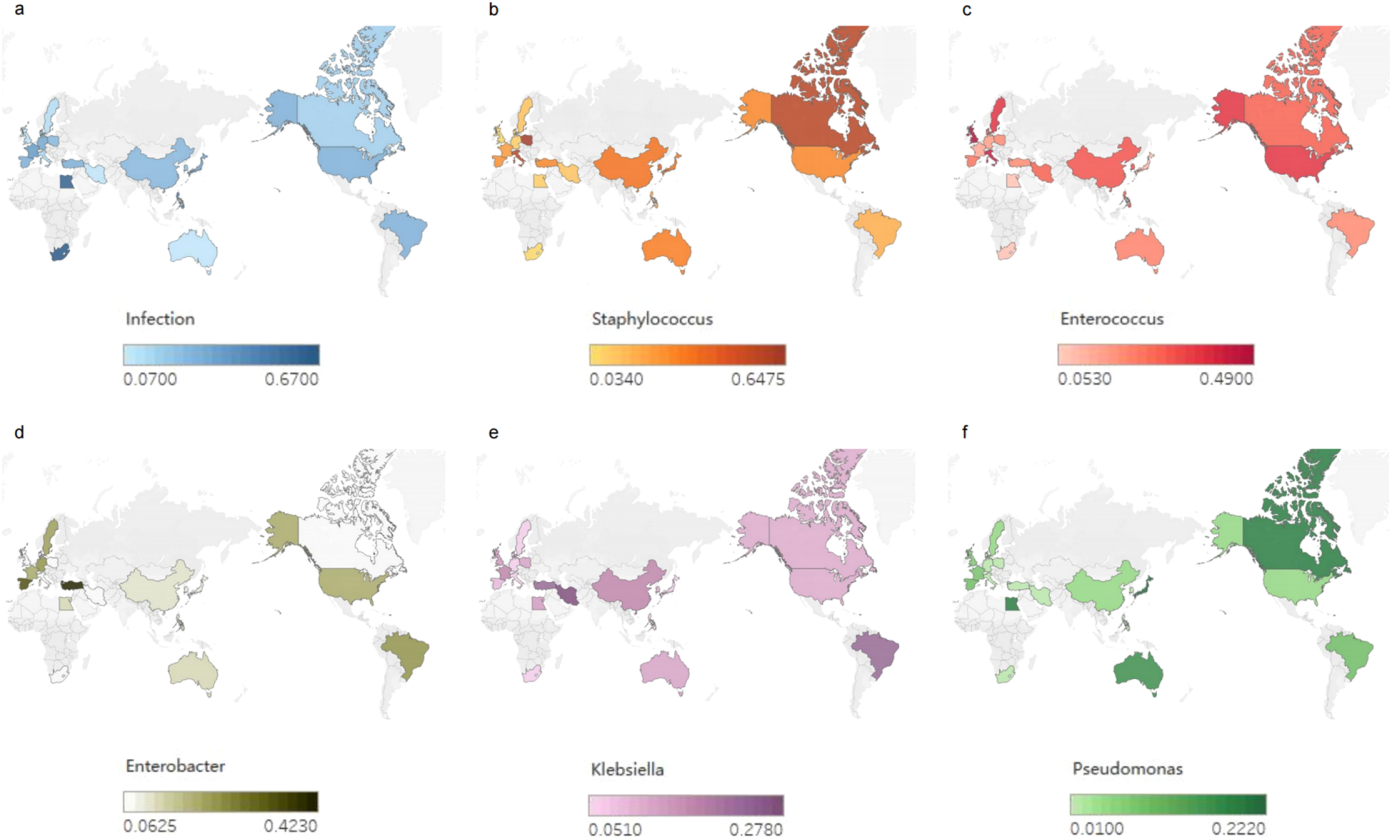
Global bacterial infections after liver transplantation (LT). **(a)** Global incidence of bacterial infections after LT. **(b-f)** Global distribution of infection rates of *Staphylococcus, Enterococci, Enterobacteriaceae, Klebsiella* and *Pseudomonas* after LT.

In Africa, gram-negative bacteria are predominant, with *P. aeruginosa* being the most common species (Mukhtar et al., 2014; Montasser et al., 2017), followed by *Klebsiella* spp. Among the gram-positive bacteria, MRSA is predominant (**Figure 1**).

Overall, infections caused by gram-negative bacteria, including *E. coli*, are relatively common, with the incidence of these infections increasing (Hand and Patel, 2016; Resino et al., 2016; Anesi et al., 2018; Jafarpour et al., 2020).

Notably, gram-negative bacteria were the predominant pathogens present during the first few decades after the advent of LT (Kusne et al., 1988; del Pozo, 2008; Martin-Gandul et al., 2015; Hand and Patel, 2016; Kritikos and Manuel, 2016; Idossa and Simonetto, 2017). Gram-positive bacteria, particularly *S. aureus* (Singh et al., 2000; Torre-Cisneros et al., 2002) and “vancomycin-resistant Enterococci” (VRE) (Newell et al., 1998), emerged as increasingly common pathogens in the mid-1990s because of technical surgical advances and catheter-related bloodstream infections (BSIs) (Singh et al., 1997). Despite the ongoing evolution of transplantation practices, gram-negative bacilli have re-emerged as the predominant bacteria causing infections after LT, with a notable increase in infections caused by MDR gram-negative bacilli (Singh et al., 2004; Garcia Prado et al., 2008; Oriol et al., 2017).

### MDR

In recent years, MDR has increased the difficulty of managing post-LT infections (Santoro-Lopes and de Gouvea, 2014; Hand and Patel, 2016; Righi, 2018). The proportion of MDR bacteria increased from less than 10% in 1998 to 23% in 2010 (Fernandez et al., 2012), and in the past five years, the infection rate has further increased to 14.3% to 47% (Freire et al., 2021; Brigati et al., 2023). Santoro-Lopes (Santoro-Lopes and de Gouvea, 2014) reviewed MDR pathogens that are prevalent in global LT centers and highlighted the close association between MDR bacterial infections and higher mortality rates. Studies indicate that gram-negative bacilli are more common and exhibit greater resistance (Zhong et al., 2012; Santoro-Lopes and de Gouvea, 2014), with postoperative *E. coli* and *K. pneumoniae* strains causing postoperative infections producing extended-spectrum beta-lactamases at rates of 59.4% and 62.1%, respectively (Zhou et al., 2006; Romero and Razonable, 2011). P*. aeruginosa* is resistant to broad-spectrum antibiotics such as cefepime, piperacillin/tazobactam, and imipenem at rates of 38%, 27%, and 14% (Mulugeta et al., 2017), respectively. Common MDR bacteria include VRE, MRSA, and “carbapenem-resistant Enterobacteriaceae” (CRE) (Kritikos and Manuel, 2016; Resino et al., 2016). The respective colonization rates, infection rates, and mortality rates are shown in **Table 1** (Santoro-Lopes and de Gouvea, 2014; Idossa and Simonetto, 2017), illustrating the growing challenges associated with managing immunosuppressed patients. These strains can cause complications such as poor wound healing and pulmonary, abdominal, urinary tract, and bloodstream infections in 20%-40% of patients postoperatively and can even cause sepsis (Kim, 2014; Santoro-Lopes and de Gouvea, 2014).

**Table 1:** Multidrug-resistant bacteria after liver transplantation (LT): colonization rate, infection rate, mortality rate, drug resistance and treatment.

Pathogen	Colonization rate (%)	Infection rate (%)	Mortality rate (%)	Drug resistance	Treatment is preferred	Secondary
ESBLE	4.1-40.6	5.5-19.6	12.9-57.4	ESBL has the capacity to hydrolyze penicillins, cephalosporins (e.g., cefotaxime, ceftazidime, ceftriaxone), and monobactams (e.g., aztreonam).	Severe infections: Carbapenems (ertapenem, meropenem, and imipenem) are the preferred treatment for mild to moderate infections. A combination of beta-lactamase inhibitors, such as cefoperazone-sulbactam, and fosfomycin, which is utilized for the treatment of urinary tract infections, is employed.	Oral fluoroquinolone or Trimethoprim-Sulfamethoxazole
CRE	6.5-33.3	2.3-29.6	5.2-34.5	Carbapenemase-free	1. Apparent sensitivity to meropenem and imipenem (i.e., minimum inhibitory concentration ≤1mg/L), but insensitive to ertapenem (i.e., minimum inhibitory concentration ≥1mg/L); extended infusion of meropenem (or imipenem-cilastatin); 2. Infections caused by Enterobacter analytes that are not susceptible to any carbapenems: cefepime/avibactam, meropenem-faborbactam, imishipenem-cilastatin-relaibactam	Eravacycline, tigecycline
				Carbapenemase-producing (enzyme type known)		
				KPC-producing	Ceftazidime/avibactam, meropenem-vaborbactam, imipenem-cilastatin-relbactam	Cefiderocol, eracycline, tigecycline
				OXA-48-producing	Ceftazidime/Avibactam	Cefiderocol, eracycline, tigecycline
				NDM-producing/Metalloamidase producing	Ceftazidime/avibactam + aztreonam is preferred; The second is cefdidil	Eracycline, tigecycline
MDRPA	2.0-3.0	9.1-15.2	25.7-42.0	Resistant to β-lactams, quinolones, aminoglycosides, and polymyxins	Choice of agent based on susceptibility and site of infection: Cefuroxime-tazobactam, ceftazidime-avibactam, imipenem-rele	Cefiderocol
CRAB	0.3-11.6	2.8-45.9	8.2-17.9	Resistant to other β-lactams, aminoglycosides, and quinolones. No standard treatment regimen is recommended.	Cefiderocol 2 g iv q8h (extended infusion > 3 hours) (for moderate to severe infections, combined with a sensitive antimicrobial	High-dose ampicillin-sulbactam + minocycline 200 mg iv/po bid + high-dose meropenem 2 g iv every 8 hours (extended infusion > 3 hours)
VRE	9.0-51.6	2.0-36.8	8.2-16.2	Resistant to vancomycin, ampicillin, penicillin G, gentamicin (highly resistant)	Enterococcus faecium, systemic infection, bacteremia: Daptomycin 10-12 mg/kg iv qd + (AMP 2g iv q4h or ceftaroline 600 mg iv q8h) Can be tried: Linezolid 600 mg po/iv ql2h or quinupristin-dalfopristin 7.5 mg/kg iv (central vein) ± AMP 2g iv q4h Note: Quinupristin-dalfopristin is only used for Enterococcus faecium.	Enterococcus faecalis: Ampicillin or penicillin resistance is rare. If aminoglycoside resistant: AMP 2g iv q4h + ceftriaxone 2g iv q12h. If penicillin resistant due to β-lactamase production: daptomycin 8 ~ 12mg/kg iv q12h + ampicillin-sulbactam 3g iv q6h
MRSA	5.9-25.2	10.3-24.2	9.9-22.3	Resistant to vancomycin (VISA or VRSA) and all beta-lactams (except ceftaroline)	Pneumonia: Vancomycin iv or Linezolid iv Bacteremia or suspected endocarditis or septic shock from bacteremia: Vancomycin 30∼60mg/kg/d, administered 2~3 times daily, target AUC24 400~600ug•h/ml Daptomycin 8~12mg/kg iv q24h	Telavancin 10 mg/kg iv once a day or linezolid 600 mg iv or po every 12 hours

ESBLE, extended-spectrum beta-lactamase producing Enterobacteriaceae; CRE, carbapenem-resistant Enterobacteriaceae; MDRPA, multidrug resistant Pseudomonas aeruginosa; CRAB, carbapenem-resistant Acinetobacter baumannii; VRE, vancomycin-resistant Enterococci; MRSA, methicillin-resistant Staphylococcus aureus.

### CRE

Since the initial identification of CRE approximately 15 years ago, the prevalence of CRE has increased worldwide. Owing to the clinical impact of numerous hospital outbreaks, especially in highly complex environments and intensive care unit (ICU) settings, CRE colonization and infections have represented some of the most investigated types of infections in transplant patients over the last decade. The overall crude mortality rate range for CRE infections in liver transplant recipients spans from a lower limit of 19.2% for 30-day mortality to a higher limit of 71% to 78% for overall mortality. In specific clinical settings such as intensive care units (ICUs), the mortality rate can rise as high as 82% (Kalpoe et al., 2012; Mularoni et al., 2019; Chen Y. et al., 2020; Liu et al., 2022). The emergence of CRE is related to multiple mechanisms, including carbapenemase production, efflux pump hyperexpression, and porin inactivation (Eichenberger and Thaden, 2019; Garcia-Bustos et al., 2022). Carbapenemases are a heterogeneous group of β-lactamases that hydrolyze carbapenems (Lynch et al., 2021; Dolci et al., 2023).

Epidemiological studies of CRE colonization and infection in LT recipients are summarized in **Table 1**. A steady increase in infections due to CRE has been documented over the past decade (Chen et al., 2021; Miller and Arias, 2024; Chang et al., 2025).

In the United States, infections due to CR-*K. pneumoniae* constitute up to 2.5% of the total infections (Wilson et al., 2017). As of 2020, most Northern and Western European countries have CR-*K. pneumoniae* infection rates of <1%. However, significantly higher rates have been reported in Southern and Eastern Europe, with rates of CR-*K. pneumoniae* infection exceeding 50% in Belarus, Georgia, Greece, Moldova, Russia and Ukraine. From 2013 - 2016, the incidence of CR-*K. pneumoniae* in Latin America ranged from 0.8 to 12.7%, and that in the Asia–Pacific region ranged from 0 to 9.3% (Castanheira et al., 2019).

Risk factors for acquiring CRE infection include prior CRE colonization, exposure to broad-spectrum antimicrobials, admission to the ICU, mechanical ventilation, prolonged hospital stay and indwelling urinary catheters (Palacios-Baena et al., 2021).

Fortunately, the treatment landscape for CRE is changing owing to the availability of newer β-lactam–β-lactamase inhibitor (BL–BLI) combinations (ceftazidime–avibactam, meropenem–vaborbactam, imipenem–cilastatin–relebactam) that target class A serine β-lactamases (including ESBLs and carbapenemases), AmpC enzymes and, in the case of avibactam, some class D OXA-48-like enzymes; the likely introduction of broad-spectrum BL–BLI combinations that inhibit or have activity against strains producing common class B metallo-β-lactamases (cefepime–taniborbactam, avibactam–aztreonam and cefepime–zidebactam, among others); and the introduction of the siderophore cephalosporin cefiderocol.

Retrospective clinical data suggest that the newer BL–BLI combinations offer a significant therapeutic advantage over polymyxin-based regimens, with lower 30-day in-hospital mortality (9% versus 32%, when comparing ceftazidime–avibactam and colistin for CRE infection) (van Duin et al., 2018; Lenhard et al., 2019). Similarly, the 30 - day mortality rates for BSIs due to CRE were reported to be 38–45%, largely before the introduction of these agents, but more recent clinical data show lower rates of mortality (34%) (Gutierrez-Gutierrez et al., 2017; Falcone et al., 2020; Perovic et al., 2020; Wang M. et al., 2022). This finding suggests that clinical outcomes for CRE infections may improve with the use of novel therapeutics.

## Bacterial infections at different sites

**Figure 2:**
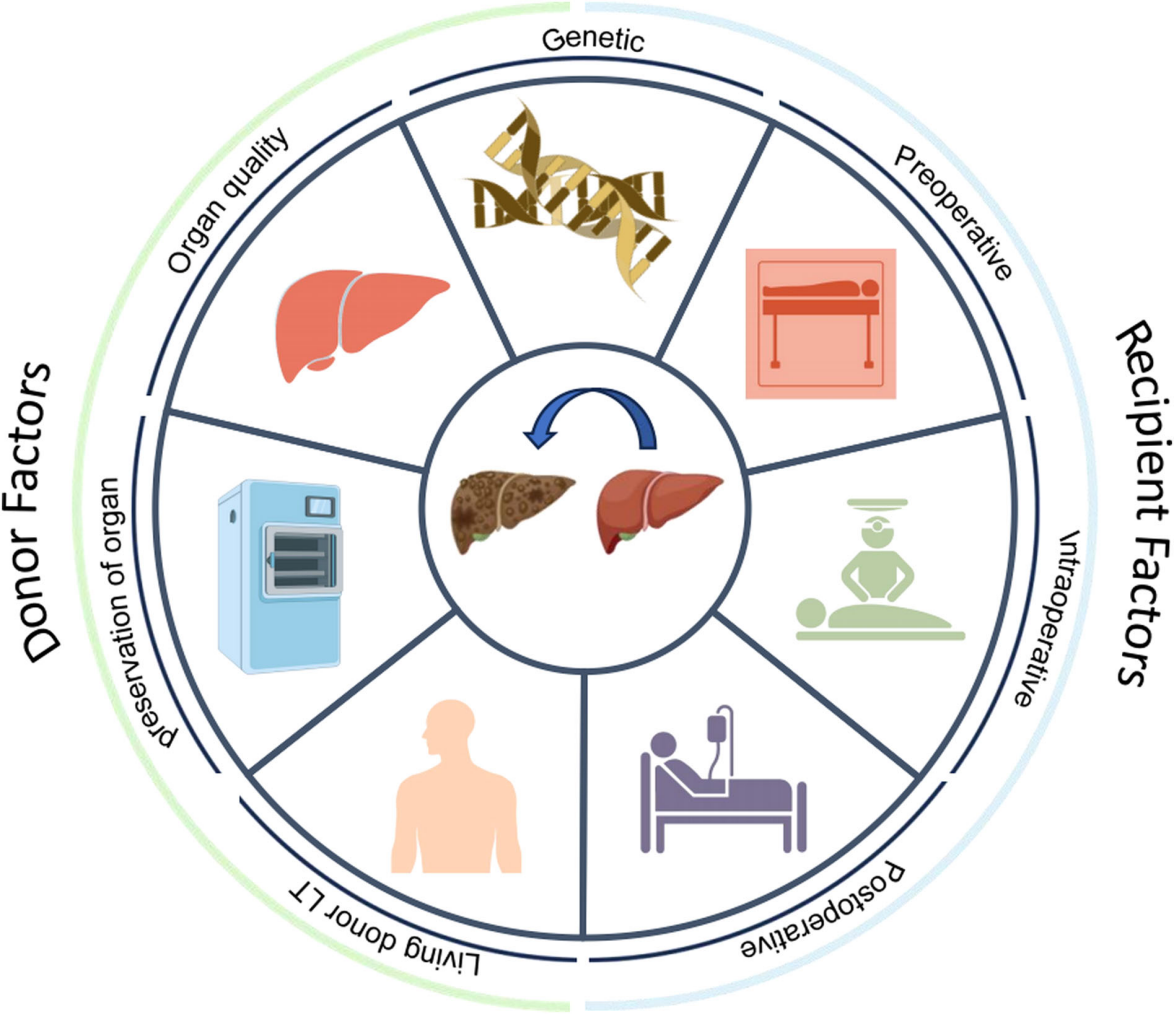
Primary sites and distribution of major pathogenic bacteria infections after liver transplantation (LT).

Bacterial infections commonly affect the surgical site (peritoneal cavity), lungs, bloodstream, biliary tract, urinary tract, and central nervous system after LT (Paya and Hermans, 1989; Blair and Kusne, 2005; Kawecki et al., 2009; Romero and Razonable, 2011). The increasing prevalence of MDR infections has further complicated the treatment of SSIs (Hand and Patel, 2016) (**Figure 2**).

### SSIs

The overall incidence of SSIs following LT ranges from 18% to 37%. Specifically, the incidence of incisional infections is between 9% and 21.5%, that of cholangitis ranges from 6% to 18%, that of peritonitis ranges from 6.3% to 9%, and that of intra-abdominal abscesses ranges from 4% to 12.9%. Severe abdominal infections are commonly caused by *E. coli* (accounting for approximately 40% of total infections) or a mixture of *E. coli* and other bacteria (Winterbottom and Jenkins, 2017; Oliveira et al., 2019; Hrenczuk et al., 2020; Schreiber et al., 2025). *Enterococcus* spp. (accounting for approximately 25% of total infections) and *S. aureus* (accounting for approximately 15% of total infections) (Meije et al., 2014) are also predominant pathogens.

Compared with patients who undergo other abdominal surgeries, LT patients are more susceptible to severe abdominal infections (Cai et al., 2023; Rodriguez-Fernandez et al., 2024). The mortality rate of sepsis due to SSIs can reach 20%-30% (Santoro-Lopes and de Gouvea, 2014). The primary causes include postoperative immunosuppression (Hashimoto et al., 2008; Kim, 2014; Pouladfar et al., 2017), prolonged peritoneal exposure during LT, gut barrier dysfunction leading to bacterial translocation, large surgical wounds causing abdominal blood seepage and accumulation, rupture of the hollow viscera due to resection of diseased liver tissue, and biliary anastomotic complications inducing abdominal fluid accumulation and bile leakage. Furthermore, the vicious cycle of low blood volume and fluid accumulation during LT can lead to acute abdominal compartment syndrome, causing a rapid increase in intra-abdominal pressure, exacerbating symptoms of abdominal infection, and further worsening the patient’s condition (Soong et al., 2012; Unal et al., 2013).

## Respiratory tract infections

Pulmonary infections rank second only to abdominal infections, with incidence ranging from 10% to 20% (Golfieri et al., 2000; Santoro-Lopes and de Gouvea, 2014; Angarita et al., 2017). Early postoperative bacterial pneumonia in LT patients is predominantly hospital-acquired, accounting for 50% to 75% of all pulmonary infections (Kim, 2014). The main pathogens causing bacterial pneumonia include *P. aeruginosa* (approximately 30%) (Angarita et al., 2017; Yamazhan et al., 2020), *Streptococcus pneumoniae* (approximately 20%), and *S. aureus* (approximately 15%), and other bacteria, as well as *E. coli*, *K. pneumoniae*, and *A. baumannii (*Angarita et al., 2017). Community-acquired pneumonia is most common more than 6 months after transplantation (Idossa and Simonetto, 2017), with the common pathogens including *S. pneumoniae*, *Haemophilus influenzae*, and *Legionella* spp (Angarita et al., 2017; Idossa and Simonetto, 2017; Dendle et al., 2018).

The main independent risk factors for postoperative pneumonia include prolonged mechanical ventilation, an extended ICU stay, postoperative pleural effusion (Shen et al., 2007), and the use of corticosteroids for acute rejection treatment (Mermel and Maki, 1990; Wu et al., 2019). The primary complication of pulmonary infection following LT is acute respiratory distress syndrome, which is a major factor contributing to the increased risk of postoperative mortality (Kim, 2014).

### BSIs

BSIs are typically associated with multiple factors, including prolonged central venous catheterization (Kim, 2014), postoperative biliary obstruction, vascular complications (Patel and Huprikar, 2012; Kim et al., 2013; Righi, 2018), and reoperation after transplantation (Chueiri Neto et al., 2019).

The incidence of BSI following LT ranges from 5% to 37% (Righi, 2018; Heldman et al., 2019). Compared with patients who undergo kidney, heart, or lung transplantation, LT recipients have a greater incidence of BSIs (Kim, 2014; Righi, 2018). Recent studies have revealed that gram-negative bacteria are more commonly responsible for bacteremia (Bert et al., 2010; Kritikos and Manuel, 2016; Idossa and Simonetto, 2017; Righi, 2018), with *Enterobacteriaceae* being the predominant pathogens.

BSIs not only prolong the length of hospital stay but also serve as predictors of the mortality and long-term survival in some LT patients (Wade et al., 1995; van Hoek et al., 2012; Meije et al., 2014; van Delden, 2014). The mortality rate can reach 40% when bacteremia is complicated by sepsis (Kawecki et al., 2014), and it can reach 50% in patients with septic shock (Kim, 2014; Pedersen and Seetharam, 2014).

## Biliary tract infections

BTIs have become one of the most common complications after LT, with an incidence rate of up to 10%-30% (Nemes et al., 2015; Wu et al., 2022). In the early post-LT period, gram-negative bacteria are the primary pathogens causing BTIs, with *E. coli* and *P. aeruginosa* being dominant. However, in the late post-transplantation period, gram-positive bacteria, particularly *E. faecalis* and *E. faecium*, become more prevalent (van Delden, 2014).

The vulnerability of the biliary system makes it prone to complications such as strictures or bile leaks, which are key factors in the development of BTIs. Post-LT biliary strictures can be anastomotic or nonanastomotic, with the latter being more common and often associated with ischemic injury (Seehofer et al., 2013). Additionally, Roux-en-Y biliary–intestinal anastomosis, a type of biliary reconstruction procedure, is associated with a greater risk of biliary tract infection than end-to-end anastomosis (Yamamoto et al., 2015). T-tube drainage or surgical catheterization during the transplant procedure may also increase the risk of infection, especially when biliary drainage is poor or catheterization is prolonged (Wojcicki et al., 2008).

The mortality rate due to BTIs after LT ranges from 15% to 40% (Cockbain et al., 2010; Kim, 2014; Resino et al., 2016; Wu et al., 2022). Patients with primary biliary cholangitis and primary sclerosing cholangitis have a greater risk of BTI after LT, as evidenced by a significantly increased postoperative mortality rate of 20-40% (Mayo Moldes et al., 2005; Fagiuoli et al., 2014).

## Urinary tract infections

The incidence of UTIs following LT typically ranges from 12.9% to 36.8% (Pouladfar et al., 2017; Jafarpour et al., 2020; Yamazhan et al., 2020). The pathogens responsible for post-LT UTIs are predominantly gram-negative bacteria (63.3%), with *E. coli* being the most common pathogen, accounting for 30.4% of all infections (Kim, 2014; Meije et al., 2014; Pouladfar et al., 2017). K*. pneumoniae* (19.1%) and *A. baumannii* (11.4%) are also significant pathogens (Kawecki et al., 2011). Among gram-positive bacteria, *Enterococcus* spp., particularly vancomycin-resistant *Enterococcus* spp., account for 17.6% of all gram-positive bacterial infections (Kawecki et al., 2011; Kim, 2014).

Catheter use during surgery and prolonged postoperative catheterization are significant risk factors for UTIs. Catheters can serve as conduits for bacterial entry infections. Additionally, owing to their shorter urethra, female patients are at greater risk of bacterial entry into the bladder, thereby increasing the risk of infection (Vidal et al., 2012; Tawab et al., 2017). Moreover, patients with a high body mass index may experience increased intra-abdominal pressure, which can contribute to the development of postoperative infections (Pouladfar et al., 2017).

UTIs in LT patients can significantly increase the risk of sepsis and renal failure, thereby increasing hospitalization and mortality rates (Rice and Safdar, 2009; Santoro-Lopes and de Gouvea, 2014; Ferrarese et al., 2018; Jafarpour et al., 2020).

## Central nervous system infections

Although CNS infections are less common (5% to 10%), they can be severe in LT patients because of the immunosuppressed state of these patients (Campagna et al., 2010; Vizzini et al., 2011; Weiss and Thabut, 2019). The initial signs of a CNS infection may be masked in these patients (Amodio et al., 2007). The mortality rate for CNS infections is extremely high (40–100%), significantly impacting patient prognosis (Feltracco et al., 2010). In post-LT CNS infections, while fungi and viruses are common pathogens, bacterial infections remain a major source of concern (Amodio et al., 2007; Feltracco et al., 2010; Weiss and Thabut, 2019). Particularly in the early postoperative period, bacterial infections are often related to the surgical technique used. Feltracco et al. noted that bacterial infections are closely associated with postoperative intra-abdominal infections, BSIs, and catheter-related infections. Common bacteria include gram-positive organisms such as *S. aureus (*Feltracco et al., 2010) and *Enterococcus* spp., as well as gram-negative bacteria such as *E. coli (*Saner et al., 2007) and *P. aeruginosa (*Feltracco et al., 2010). These bacteria can invade through catheters, surgical sites, or the respiratory tract and are then disseminated to the CNS. *Listeria monocytogenes* is often transmitted through contaminated food sources such as milk, cheese, undercooked meat, and vegetables (Sartor et al., 2015). In the later stages of transplantation, meningitis can also be secondary to *Mycobacterium tuberculosis* infection (Weiss and Thabut, 2019).

## Risk factors

### Clinical aspects

#### Donor factors

**Figure 3:**
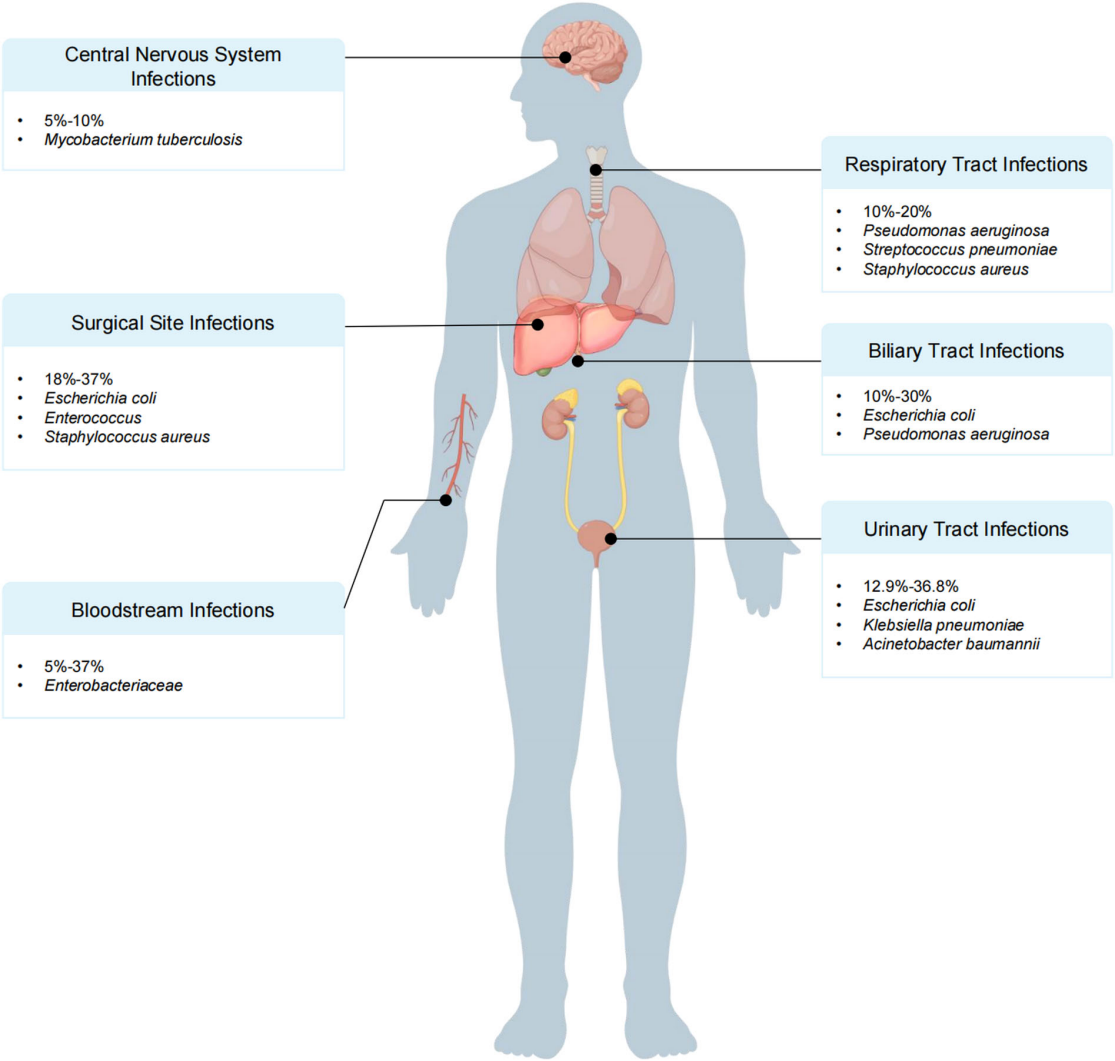
Donor and recipient risk factors for bacterial infection after liver transplantation (LT).

The incidence of infection in donors can reach 19.2% (Nanni Costa et al., 2008). The spectrum of donor-derived infections after LT was dominated by gram-negative bacteria, among which *K. pneumoniae*, *E. coli*, and *P. aeruginosa* were the most common pathogens. It is important to acknowledge the substantial impact of the prevalence of carbapenem-resistant strains on clinical prognosis, particularly in the context of carbapenem-resistant *K. pneumoniae*, carbapenem-resistant *P. aeruginosa*, and “carbapenem-resistant Acinetobacter baumannii” (CRAB). Furthermore, MRSA has emerged as a significant pathogen in cases of donor-derived infection. The infection of the aforementioned MDR bacteria not only increases the difficulty of anti-infective treatment but also affects the survival rate of patients (Altman et al., 2014; Giani et al., 2014; Miller et al., 2015; Lewis and Sifri, 2016; Ye et al., 2017; Tong et al., 2020; Song ATW. et al., 2024) (**Figure 3**).

The donor factors primarily involve the quality, maintenance and preservation of organs. Livers from donors with moderate-to-severe fatty liver disease (Xie et al., 2024) and from those of advanced age may increase the risk of postoperative infection in recipients (van Hoek et al., 2012; Reddy et al., 2017). Additionally, a history of diabetes and liver dysfunction in the donor can also affect the rate of bacterial infections after LT. Urrunaga et al. conducted an analysis of the UNOS database and reported that all cohorts of older donors over 50 years of age presented increased risks of morbidity and mortality from sepsis (Lee et al., 2023). An analysis involving 129 patients revealed that donor age was associated with the infection incidence, with an average donor age of 36 years in the infection group and 30.5 years in the infection-free group (Gayowski et al., 1998).

With respect to the quality of organ maintenance and preservation, donors who have spent three or more days (Corman Dincer et al., 2019) in the ICU, who require vasoactive drugs to maintain blood pressure, who have undergone cardiopulmonary resuscitation, or who have experienced prolonged cold/hot ischemia are at a significantly greater risk of infection (Grossi et al., 2009; Ison and Nalesnik, 2011; van Hoek et al., 2012; Reddy et al., 2017; Wu et al., 2022).

Among these factors, living-donor LT results in greater susceptibility (11% vs. 4%) (Freise et al., 2008; Samstein et al., 2016) to biliary infections than does deceased-donor LT, which is attributable to the unique procedure. First, small donor grafts may lead to postoperative liver dysfunction with prolonged cholestasis and coagulopathy. Second, biliary leakage from the cut surface of the graft may subsequently lead to biloma formation, which may lead to secondary infection. Third, the surgical procedure for living-donor LT is technically more challenging and contributes to a higher incidence of complications such as biliary strictures (Kiuchi et al., 1999; Tucker and Heaton, 2005; Liu et al., 2006). *Enterococcus* spp. were the most frequently isolated pathogens (31%), followed by *E. coli* (15%) (Kim et al., 2008; Li C. et al., 2012; Pedersen and Seetharam, 2014; Abad et al., 2017).

#### Recipient factors

##### Preoperative factors

Preoperative risk factors primarily include the patient’s underlying disease and immunity status. Studies have shown that comorbidities such as cirrhosis, diabetes, chronic kidney disease, and a high MELD score (>20) increase the risk of postoperative infection (Pouladfar et al., 2017; Eklou et al., 2022). A history of surgery, severe obesity or cachexia, preoperative mechanical ventilation, and infection or antibiotic treatment within two months prior to surgery can also increase the risk of postoperative infection (Kim, 2014; Ying et al., 2020; Wu et al., 2022). These conditions can lead to a weaker immune response, thereby increasing the incidence of postoperative bacterial infections (Avkan-Oguz et al., 2013).

##### Intraoperative factors

Prolonged surgery duration (>8 hours), significant blood loss (>1000 ml), and extensive blood transfusion (erythrocytes > 6 U) (Shen et al., 2007; Li C. et al., 2012; Wu et al., 2019) are significantly associated with postoperative infections. These factors can lead to immunosuppression and tissue hypoxia (Velusamy et al., 2020), as well as gut flora translocation, and prolonged surgical exposure increases the opportunity for bacterial entry. Additionally, intraoperative management directly affects postoperative infections, with procedures such as common bile duct jejunostomy being risk factors for infection (Rayes et al., 2005; Pedersen and Seetharam, 2014; Anesi et al., 2018).

Biliary reconstruction remains a major point of controversy, particularly in living donor LT. The major concerns are early leakage and late stricture at the anastomotic site, which are associated with technical, anatomical, or microcirculatory considerations. Early biliary complications may lead to a fatal outcome in recipients who are critically ill or who receive relatively small grafts, and these conditions themselves may increase the risk of complications (Braun et al., 2021).

##### Postoperative factors

Patients are in an immunosuppressed state after LT. Factors such as an ICU stay of 9 days or more (Li C. et al., 2012; Laici et al., 2018; Jafarpour et al., 2020), the use of broad-spectrum antibiotics (Zhong et al., 2012), continuous positive pressure ventilation for more than 12 hours (Shen et al., 2007), prolonged catheterization (including specific endotracheal intubation, deep venous cannulation, and an abdominal drainage tube), and postoperative reoperation can expose patients to infection sources for extended periods, ultimately increasing the risk of infection (Wade et al., 1995; Avkan-Oguz et al., 2013; Selimoglu et al., 2020) (**Figure 3**).

##### Genetic factors

Genetics influence a recipient’s postoperative immune status and play a crucial role in susceptibility to bacterial infections following LT. Numerous studies have revealed that polymorphisms in the Toll-like receptor 2 (TLR-2) (Lee et al., 2011a), Toll-like receptor 9 (TLR-9) (Tsujimoto et al., 2006; Carrera-Silva et al., 2008; El-Bendary et al., 2020), mannose binding lectin 2 (MBL2), ficolin 2 (FCN2), and mannan-binding lectin serine protease 2 (MASP2) genes in donors and recipients (de Rooij et al., 2010; de Rooij et al., 2011; Igarashi et al., 2014; Debette-Gratien et al., 2016), the SLCO1B1 rs4149015 AA genotype in recipients (Guo et al., 2025), as well as interleukin-10 (IL-10) polymorphisms, may affect the expression levels of these genes and thereby alter the patient susceptibility to bacterial infections (Chuang et al., 2009; Shi et al., 2017; Dai et al., 2020).

The polymorphism of the C7 rs6876739 gene in donors is associated with the risk of early bacterial infections post-LT (Shen et al., 2012; Zhong et al., 2016). This phenomenon is related to the C7 protein and the generation of the membrane attack complex, which plays a role in antimicrobial processes by activating the NLRP3 inflammasome and releasing IL-1β (Shen et al., 2012; Zhong et al., 2016; Wu et al., 2019). Additionally, IL-18 stimulates the production of interferon-γ by natural killer cells and T cells to impact the occurrence of postoperative infection. One of its primary functions is to stimulate the production of interferon-γ by natural killer cells and T cells, which is crucial for the regulation of innate and adaptive immune responses (Kinoshita et al., 2004; Ghose et al., 2011; Ono et al., 2012). In experiments with a mouse burn model, IL-18 knockout mice presented a 35% reduction in infection rates (Kinoshita et al., 2006). Research has confirmed that the variation in the IL-18 rs1946518 genotype is significantly associated with an increased infection risk (Shi et al., 2017).

Pannexin-1 (Panx1) has been reported to be a key immunoregulatory target in post-transplantation infections. Panx1 is a transmembrane protein that allows signaling molecules such as extracellular adenosine triphosphate to be released from inside the cell to the extracellular space, thereby activating various downstream signaling pathways, including those associated with P2 receptors, and regulating inflammatory responses, apoptosis, and immune responses (Li et al., 2021). Through 64 eQTL data analyses and 200 independent sample validations, the donor liver Panx1 rs79475114 CC genotype was identified as an independent risk factor for MRSA infection post-LT. Additionally, a mouse LT model of MRSA infection post-LT confirmed that donor liver Panx1-mediated adenosine triphosphate release acts on the hepatocyte membrane P2X2 receptor, recruiting neutrophils and M1 macrophages to exert an anti-MRSA infection effect (Li et al., 2021). Furthermore, in patients with sepsis caused by gram-negative bacteria, Panx1 expression in the donor liver was significantly reduced, leading to decreased IL-33 release through the inhibition of the P2X7/NLRP3 signaling pathway, which reduced the proliferation and differentiation capacity of ST2^+^ Treg cells, increasing the incidence and mortality of sepsis after LT (Wang P. et al., 2022). Notably, Panx1 not only plays a role in the early stage of acute bacterial infections but also participates in the long-term immune surveillance mechanism of the liver. In certain chronic infection scenarios, such as peritoneal infections resulting from bacterial translocation in the gut, the Panx1-regulated adenosine triphosphate–P2X pathway is crucial for maintaining local immune homeostasis (del Pozo, 2008). This finding indicates that the absence of Panx1 significantly increases the risk of post-LT infections, particularly drug-resistant bacterial infections.

#### Diagnostic approaches

Diagnosing infections in transplant recipients is more challenging than in individuals with normal immune function, as the symptoms and signs of infection are often ignored. Additionally, transplant recipients may exhibit noninfectious causes of fever, such as allograft rejection, necessitating further exclusion.

#### Traditional diagnostic methods

Common diagnostic methods include imaging examination and microbiological testing.

High-resolution computed tomography (HRCT), magnetic resonance imaging (MRI) and other imaging studies can effectively aid in diagnosing peritoneal abscesses, BTIs, pulmonary infections, urinary tract obstructions, and CNS infections as potential primary infection sources (Feltracco et al., 2010; Vizzini et al., 2011; Qin et al., 2012; Kim et al., 2013). Additionally, magnetic resonance cholangiopancreatography is widely used to detect biliary strictures and leaks and allows noninvasive evaluation of the anatomy and function of the biliary system (Wojcicki et al., 2008).

Blood or body fluid cultures remain the gold standard for diagnosing infections. Blood cultures can be used to determine the causative pathogens of BSIs (Bert et al., 2010; Kawecki et al., 2011; Abad et al., 2017). For patients diagnosed with or highly suspected of having a BTI, bile cultures are used to identify the causative pathogens. Urine cultures are the primary method for diagnosing UTIs, allowing the detection of pathogenic species (Kawecki et al., 2011; Pouladfar et al., 2017). Cerebrospinal fluid analysis, including bacterial, fungal, and viral cultures of cerebrospinal fluid, is key in diagnosing CNS infections, and susceptibility testing is key for selecting appropriate antibiotics (Guo et al., 2022).

#### Novel diagnostic techniques

In recent years, with the advancement of molecular biological technologies, an increasing number of novel diagnostic methods have been applied to detect bacterial infections after LT. These techniques include digital droplet PCR (ddPCR), real-time PCR combined with high-resolution melting (HRM) curve analysis, nanoprobes with surface-enhanced Raman spectroscopy (SERS), and cell-mediated immunity.

#### ddPCR technology

ddPCR involves dividing the sample into tens of thousands of microdroplets, with individual PCR amplification being performed for each droplet. The principle involves precise detection of low-abundance DNA sequences, making it particularly suitable for the early detection of low-level bacterial infections. Compared with traditional PCR, ddPCR offers more precise quantitative assessment (Caviglia et al., 2018; Liu et al., 2024). Mirna et al. used ddPCR to detect and quantify *A. baumannii* in mini-BAL fluid and, by comparing colonization data, quantitative culture results, and different generations of PCR, showed that ddPCR is more sensitive than other molecular techniques (Giselle Moreira et al., 2024).

Although ddPCR provides absolute quantification, theoretically reducing interlaboratory variability, variability still exists. Factors ranging from differences in nucleic acid extraction methods to variations in primer-probe design, as well as the stability of droplet generation processes, can all influence the final results (Ramanan and Razonable, 2013). A major barrier to the adoption of ddPCR technology is its economic cost. Both the initial equipment investment for ddPCR systems (such as Bio-Rad QX200 and QIAGEN QIAcuity) and the reagent and consumable expenses per test typically exceed those of established qPCR workflows (Ai et al., 2024). Although some literature suggests ddPCR may offer potential cost-effectiveness advantages (Kojabad et al., 2021), the prevailing view in clinical adoption is that its high cost remains the primary barrier to widespread implementation (Ai et al., 2024).

### High-resolution melting curve analysis

Real-time PCR combined with high-resolution melting curve analysis utilizes the melting characteristics of 16S rRNA gene amplification products to distinguish between various bacterial species precisely. This technique has shown significant advantages in the rapid diagnosis of peritoneal infections and BTIs, particularly in the case of acute infection requiring rapid intervention (Cheng et al., 2006; Hu et al., 2014). This method has partially replaced traditional culture methods for improved detection efficiency, but it requires high-precision instruments. Future optimization through algorithm improvements could increase the data processing speed, facilitating larger-scale clinical applications.

Although the reagent costs for HRM are relatively low, this technology requires a real-time quantitative PCR instrument equipped with high-resolution melting functionality and specialized analysis software (Verweij and Stensvold, 2014). More importantly, accurately interpreting subtle differences in melting curves requires extensive experience and well-established interpretation criteria—qualities not readily available in all clinical laboratories (Xu et al., 2021).

#### Nanoprobes and SERS detection

Nanoprobe technology achieves ultrasensitive detection of bacteria through SERS signals (Kearns et al., 2017; Cialla-May et al., 2024). Gao et al. introduced a SERS nanoprobe, AMD@HA, which utilizes localized surface plasmon resonance for photothermal therapy and enhanced Raman signals, providing a sensitive and noninvasive diagnostic tool for the rapid identification and eradication of MDR bacteria (Gao et al., 2024). Currently, SERS is still in the promotional stage in clinical settings, but its specificity and stability are evident. In the future, by improving the specificity of the nanoprobe structure, SERS could become an important high-throughput detection tool in clinical practice.

The entire workflow of SERS, from sample pretreatment and synthesis of nanoscale substrates to spectral acquisition and data post-processing, severely lacks standardized operating procedures (Tadesse et al., 2020). SERS spectra are typically highly complex and contain a wealth of information, often requiring sophisticated machine learning or chemometric algorithms for pattern recognition and interpretation. This makes it difficult to directly compare results across different laboratories (Tadesse et al., 2020). The lack of universally accepted standardized protocols and certified reference materials poses a significant barrier to obtaining regulatory approval and advancing into clinical applications (Cialla-May et al., 2024). Although the cost of precious metal raw materials (gold, silver) required for a single test may not be high, the cost of manufacturing highly uniform, reproducible nanostructures using advanced technologies such as photolithography is extremely high (Pelaz et al., 2017). How to economically scale up this production from the laboratory level to commercial, clinical-grade scale remains an unresolved challenge to this day (Pelaz et al., 2017).

#### Microbial cfDNA sequencing technology

cfDNA of microbial origin is released into the host’s body fluids during infection with pathogenic microorganisms (Muller et al., 2024). Free microbial DNA can be extracted from blood or body fluids and sequenced via next-generation sequencing (NGS), which can be used for the early detection of all known and unknown bacterial infections. Li et al. proposed a new multiplex PCR assay, MeltArray, which uses intact microbial cells as the source of genomic DNA (gDNA). The MeltArray assay showed a sensitivity of 93.8%, specificity of 98.6%, positive predictive value of 99.7%, and negative predictive value of 75.0%. In comparison, a cross-sectional study on the diagnostic value and clinical application of mNGS in post-liver-transplant infections reported sensitivity of 22.22%, specificity of 89.28%, positive predictive value of 66.67%, and negative predictive value of 54.35% (Zhao et al., 2022). They concluded that the MeltArray assay can be used as a rapid and reliable tool for the direct identification of pathogens in BSIs (Song J. et al., 2024). Furthermore, microbial cfDNA sequencing technology is characterized by its noninvasiveness, high sensitivity, and specificity, making it particularly suitable for detecting pathogens that are difficult to culture using traditional methods (Hennis et al., 2024).

The per-test cost of mNGS is extremely high, typically reaching thousands of dollars, which constitutes a major barrier to its routine application and a significant factor requiring careful consideration in hospital budgets (Azar et al., 2022). Although studies indicate that mNGS may reduce overall healthcare costs by shortening fever duration and optimizing antibiotic use (Jablonska et al., 2025), securing stable reimbursement from health insurance remains a challenge, significantly limiting its clinical accessibility. Currently, the analytical workflow and microbial reference databases used in mNGS lack standardized protocols, potentially leading to discrepancies in analysis results for the same sample across different laboratories (Tan et al., 2024).

#### Cell-mediated immunity

A novel global cell-mediated immunity assay (QuantiFERON Monitor [QFM], Qiagen) that measures plasma interferon-gamma (IFN-γ) levels after stimulation of whole blood with a combination of antigens designed to stimulate both the innate and adaptive arms of the immune system has been developed. A previous study using this assay revealed that plasma IFN-γ levels in liver transplant candidates and recipients were significantly lower than those in healthy control subjects (Sood et al., 2014). Kumar et al. performed a prospective observational cohort study in which a global immune monitoring assay was used to predict infectious complications during the first year post-transplantation. They reported that the assay was broadly predictive of the likelihood of subsequent infectious complications, with patients with low IFN-γ values being at the highest risk of subsequent infection. The best predictive value was observed at the 6-month time point, although predictive utility was observed at all 3 time points (Mian et al., 2018).

CMI testing is typically technically complex, labor-intensive, and costly. It often requires the use of fresh whole blood samples processed within a short timeframe after collection, placing high demands on laboratory technical capabilities and logistics (Chung et al., 2016). This limits the accessibility of CMI monitoring primarily to large transplant centers equipped with specialized immunology laboratories, making routine monitoring difficult in smaller hospitals or outpatient settings (Chung et al., 2016).

### Prevention and treatment

Measures to prevent and treat bacterial infections following LT are categorized primarily into perioperative antibiotic use and nonantibiotic preventive measures, both of which play crucial roles in the management of these infections.

#### Perioperative antibiotic use

Microbial contamination of the donor organ preservation solution should be managed through the following steps: 1. Sterile sampling and testing—the storage solution should be subjected to NGS and cultured for bacteria immediately at the time of organ acquisition. 2. Pollution treatment—if the preservation solution tests positive, specific antibiotics (such as carbapenems or tigecycline for gram-negative bacteria) should be selected for local lavage of the donor organs according to the drug sensitivity results. The donor liver should be soaked with a storage solution containing antibiotics (e.g., a UW solution containing vancomycin and fluconazole) for 30 minutes to reduce the pathogen load (Theodoropoulos et al., 2021; Lentine et al., 2023; Pouch et al., 2025).

Nearly all LT procedures require the prophylactic administration of broad-spectrum antimicrobial agents (Resino et al., 2016; Lakomkin and Hadjipanayis, 2021; Williams et al., 2021; Steffani et al., 2024). For the prevention and treatment of SSIs, measures should primarily target gram-negative bacilli. High-risk recipients should also be protected against gram-positive cocci and fungi (Kim, 2014). Combination therapy with beta-lactams and beta-lactamase inhibitors, and if necessary, vancomycin, can be used (Chueiri Neto et al., 2019). The choice of antibiotics should be adjusted on the basis of the recipient’s allergy history, pathogen identification results, and renal function (Li H. et al., 2012; Kim, 2014; van Delden, 2014). The typical duration of prophylactic antibiotic administration is 24–72 hours post-surgery (Hashimoto et al., 2007; Hashimoto et al., 2008; Hashimoto et al., 2009). For transplants from high-risk donors (such as those colonized with MDR bacteria), the recipient should receive 7–14 days of targeted anti-infection treatment after surgery. Additionally, oral administration of rifaximin has been shown to be effective at preventing post-LT infections (Neff et al., 2010; Sun et al., 2012; Salehi et al., 2019; Campos-Varela et al., 2022).

However, the misuse of antibiotics can lead to the emergence of MDR organisms, posing challenges to antibiotic prophylaxis strategies (Patel and Huprikar, 2012; Fagiuoli et al., 2014; van Delden, 2014). Once MDR infection has been diagnosed, empiric treatment must be started directly to reduce complications and mortality. The treatment of MDR infections should be guided by the type of pathogen and susceptibility testing, with a minimum treatment duration of 2–3 weeks (Bert et al., 2010; Kim, 2014). For *Enterobacteriaceae* strains that produce broad-spectrum lactamases, carbapenems are preferred, followed by lactam/lactamase inhibitors or cephalosporins. For CRE, ceftazidime-avibactam and tigecycline as monotherapy or combination therapy, or polymyxin-based combination therapy, can be considered. For metallo-beta-lactamase-producing CRE, ceftazidime-avibactam combined with aztreonam is recommended. For CRPA, combination therapy with ceftazidime–avibactam, polymyxin, or antipseudomonal beta-lactams can be used. Combination therapy with sulbactam and its derivatives, tigecycline, or polymyxin may be considered as a secondary choice. For MRSA and methicillin-resistant MRCNS, vancomycin, linezolid, daptomycin, or teicoplanin can be chosen for treatment (Silva et al., 2018). **Table 1** summarizes the antibiotics and the conditions for which each is used (Horcajada et al., 2019; Tamma et al., 2019; Heil et al., 2021; Tamma et al., 2021; Antimicrobial Resistance C, 2022; Castanheira et al., 2023; Meschiari et al., 2024; Miller and Arias, 2024; Tamma et al., 2024).

## Nonantibiotic preventive measures

### Selective decontamination of the intestine

SID is another widely used preventive measure that reduces the abundance of gram-negative bacteria in the intestine via the use of antibiotics that do not kill anaerobes, thereby reducing the incidence of infections caused by these strains. Resino et al. investigated the effectiveness of SID in preventing postoperative infections and reported that it significantly reduced the occurrence of gram-negative bacterial infections (Rayes et al., 2005). However, it may increase the risk of gram-positive infections, especially enterococcal infections (Resino et al., 2016). By selectively removing gram-negative bacteria from the intestine, SID lowers the risk of these pathogens translocating to the bloodstream or surgical wounds, thus reducing the likelihood of related infections. The advantage of this method is that it maintains the presence of anaerobes, thereby preserving the balance of the gut microbiota (Resino et al., 2016).

### Probiotics and prebiotics

Early enteral nutrition and the administration of probiotics to modulate the gut flora have been shown to reduce the incidence of infections and effectively shorten the length of ICU stay and overall hospitalization time (Wang et al., 2015; Annavajhala et al., 2019; Ancona et al., 2021; Cooper et al., 2022). Probiotics primarily exert their clinical effects by maintaining gut barrier function, promoting the colonization by beneficial bacteria, and competitively excluding harmful bacteria. Beneficial bacteria produce metabolites such as short-chain fatty acids, which enhance the intestinal epithelial barrier and inhibit the growth of pathogenic bacteria while also regulating the host’s immune response and reducing the release of inflammatory factors (Zhang et al., 2013; Sawas et al., 2015; Kahn et al., 2020). Commonly used probiotics include *Bifidobacterium* spp. and *Lactobacillus* spp., as well as *synbiotics*, which are combinations of prebiotics and probiotics. These strains have been shown in multiple clinical studies to improve the gut microecology posttransplantation, reducing the colonization and translocation of pathogenic bacteria (Rayes et al., 2005; Zhang et al., 2013; Kahn et al., 2020; Cooper et al., 2022; Yoshiya et al., 2025).

### Improvements in surgical techniques and postoperative management

Precise surgical techniques and meticulous postoperative management are crucial (Kim et al., 2019). Shorter operative times and less trauma during intraoperative procedures reduce the incidence of postoperative infections. Optimizing the surgical procedure can effectively reduce the chance of infection.

Several aspects should be considered for patient management when managing patients after LT. First, strict fluid volume control and maintenance of the lower central venous pressure are essential to prevent pulmonary edema and cardiac insufficiency while protecting liver and kidney function and avoiding infections at other sites, such as catheter-related infections, especially in patients with severe abdominal infections. Effective removal of the source of infection, such as the timely removal of central venous catheters, endotracheal tubes, and urinary catheters, is crucial for infection control (Patel and Huprikar, 2012; Zhong et al., 2012; Meije et al., 2014; Pedersen and Seetharam, 2014; Righi, 2018; Kim et al., 2021). Additionally, management should begin at the primary site of infection. For example, surgical intervention is often necessary to remove the source of infection in patients with intra-abdominal infections (Shoji et al., 2015). However, a focus on necrotic tissue and strict adherence to the principles of damage control are important. Removal of all the purulent necrotic tissue in the abdomen is not necessary, as this procedure can increase the absorption of bacteria and toxins into the bloodstream and exacerbate dysfunction of the respiratory, circulatory, and other systems. Open exploration of the lower abdomen should be avoided to prevent further contamination of the peritoneum. If a patient develops sepsis, intensive care and the use of vasopressors to maintain blood pressure are typically needed (Kawecki et al., 2009). Furthermore, for biliary infections caused by structural issues such as biliary stricture or bile leakage, ERCP, percutaneous transhepatic biliary drainage interventional therapy, or even surgical intervention may be necessary to relieve the obstruction, repair the biliary system, or remove infected bile (Nemes et al., 2015).

Finally, the use of immunosuppressants should be adjusted according to the infection status to help the body restore its own immune defense function (Wu et al., 2019).

### Immunosuppressive agents

Currently, the tacrolimus-based immunosuppressive regimen is widely used in clinical practice (Tolou Ghamari and Palizban, 2023). International recommendations suggest minimizing antirejection therapy for organ transplantation (Thomson et al., 2020). Studies have shown that maintaining the tacrolimus dose at a dose of 4–10 ng/ml can prevent acute rejection and reduce the occurrence of side effects such as infections (Shi et al., 2023). When an infection occurs, therapeutic drug monitoring is needed, and dynamic adjustments should be made in combination with the severity of the infection. If the infection continues to progress, the dosage can be further reduced, or administration can be temporarily discontinued (≤ 7 days).

Owing to the dual effects of mycophenolate mofetil (MMF) on bone marrow suppression and immunosuppression, multiple clinical studies have confirmed that discontinuing MMF during severe infections can improve infection control and that short-term discontinuation (e.g., 7–14 days) does not significantly increase the risk of rejection. Therefore, we recommend the early withdrawal of MMF when an infection occurs and a gradual increase in the dosage after the pathogen is cleared. Moreover, the lymphocyte counts and the levels of inflammatory markers (such as white blood cells, C-reactive protein and procalcitonin) should be monitored to avoid excessive immunosuppression.

Currently, most centers use antirejection regimens without corticosteroids or with low-dose corticosteroids (Massarollo et al., 1998; Shepshelovich et al., 2019; Trezeguet Renatti et al., 2024). Most studies suggest gradually reducing the steroid dose during severe infections rather than sudden withdrawal. For example, in LT patients with severe infections, the methylprednisolone dose can be gradually reduced to physiological levels (e.g., 5–10 mg/d hydrocortisone) to avoid adrenal insufficiency and the inflammatory cytokine storm caused by the sudden withdrawal of immunosuppression. The pharmaceutical agents employed in the treatment of this condition are listed in **Table 2** (Chen F. et al., 2020; Harada et al., 2020; Marx et al., 2020; Ueno et al., 2020; Wang et al., 2020; Yamada et al., 2020; Zegarska et al., 2020; Deppermann et al., 2021; Friman et al., 2021; Gilad et al., 2021; Tustumi et al., 2021; Udomkarnjananun et al., 2021; Vnucak et al., 2021; Campos-Varela et al., 2022; Cinar and Bulbuloglu, 2022; Kniepeiss et al., 2022; Rabbani et al., 2022; Wei et al., 2022; Mathew and Philips, 2023; Park et al., 2023; Amjad et al., 2024; Diaz et al., 2024; Ntobe-Bunkete and Lemaitre, 2024; Tharanon et al., 2024).

**Table 2:** Adjusting immunosuppressive medicines for bacterial infections after liver transplantation (LT).

Triple - therapy (CNIs + MMF + short - term corticosteroids) or dual - therapy (CNIs + MMF) as the postoperative basic regimen				
Drug Name	Standard Immunosuppressive Regimen	Immunosuppression Adjustment in Acute Infection Phase	Management During Recovery and Long-Term Phase	Adjustment Strategies for Special Infection Scenarios
CNIs	Target trough levels: 8-12 ng/mL in the early postoperative period, reduced to 5-8 ng/mL after 3 months.	Tacrolimus: Reduce trough levels to 4-6 ng/mL or temporarily discontinue. Cyclosporine: Adjust dose based on metabolite monitoring (e.g., OH-CsA). Elevated metabolite levels correlate with increased infection risk.	Gradual recovery scenarios: Timing: After the infection the original immunosuppressive regimen is gradually restored. CNIs are preferentially adjusted to the target trough concentration (e.g., tacrolimus 4 - 6 ng/mL). For recurrent infectors: Long - term use of low - dose CNIs in combination with mTOR inhibitors, or discontinuation of MMF to reduce the risk of recurrent infection. Dynamic monitoring indicators Drug concentration: CNIs and mTOR inhibitors need to be monitored for trough concentrations 1 - 2 times a week to optimize the dosage for precision. Immune status: Evaluate the risk of over - immunosuppression through WBC. Infection markers: CRP and PCT are used as dynamic monitoring indicators to guide the re - initiation timing of immunosuppressants.	Septic shock or septicemia Immediately suspend all non - essential immunosuppressants (MMF, mTOR inhibitors), and only retain the lowest - dose corticosteroids (such as 50 mg/day of hydrocortisone). Multidrug - resistant bacterial infection Strengthen immunosuppressive dose reduction: The trough concentration of CNIs is further reduced (tacrolimus < 4 ng/mL), combined with broad - spectrum antibiotics covering resistant bacteria.
MMF	Adult dose: 1000-3000 mg/day; pediatric dose: 30-50 mg/kg/day. Combination with CNIs may allow lower CNI dosing.	For severe infections (bacteremia, pneumonia): Preferentially suspend MMF, or reduce the dosage to 500 - 1000 mg/day.		
Corticosteroids	In the early postoperative period, a large dose is used (such as 500 mg/day of methylprednisolone), and the dosage is gradually reduced until it is discontinued within 3 months or maintained at a low dose (5 - 10 mg/day).	Maintain prednisone at 5 - 10 mg/day to avoid complete drug withdrawal and prevent adrenal crisis or rejection reactions.		
mTOR Inhibitors	When used as a substitute for CNIs, the target trough concentration is 5 - 10 ng/mL, which can reduce metabolic complications.	Substitution strategy: In severe infections, CNIs or MMF can be converted to mTOR inhibitors, but their antiproliferative effect may delay wound healing, and they are contraindicated in active abdominal cavity infections.		

CNIs, calcineurin inhibitors; MMF, mycophenolate mofetil; mTOR, mammalian target of rapamycin; WBC, white blood cell; PCT, procalcitonin.

## Conclusions

The management of MDR bacterial infections following LT presents significant challenges, especially since the current literature lacks standardized control and prevention protocols (Zhong et al., 2012; Kim, 2014). Future approaches may include the combination of broad-spectrum antibiotics with personalized medication regimens and isolation measures for high-risk patients to reduce the transmission of MDR organisms. Currently, no comprehensive strategies are available for managing postoperative infections at diverse sites, particularly the abdomen, biliary tract, and urinary tract. Large-scale studies are needed to develop a multistage, site-specific comprehensive management strategy to optimize preoperative, intraoperative, and postoperative prevention and treatment processes (Nafady-Hego et al., 2011; Taddei et al., 2023). Additionally, while bacterial infections are major postoperative complications, strategies involving the rational use of antibiotics and selective gut decontamination (Resino et al., 2016), along with variability in environments across centers, hinder the development of standard practices. In future studies, researchers should integrate multidimensional factors to further explore infection mechanisms and refine prevention and management strategies.
